# Isolating unique variance in mental health outcomes attributable to personality variables and childhood emotional abuse

**DOI:** 10.3389/fpsyg.2024.1330483

**Published:** 2024-01-22

**Authors:** Chantal Van Landeghem, Lorna S. Jakobson

**Affiliations:** Department of Psychology, University of Manitoba, Winnipeg, MB, Canada

**Keywords:** alexithymia, sensory processing sensitivity, anxiety sensitivity, childhood emotional abuse, physical activity, sex differences, COVID-19 pandemic

## Abstract

**Introduction:**

University students are at high risk for anxiety and depression. Our main objective was to tease apart variance in symptom severity that was uniquely attributable to four associated variables that are frequently confounded: exposure to childhood emotional abuse, alexithymia, sensory processing sensitivity (SPS), and anxiety sensitivity (AS).

**Methods:**

University students (*N* = 410) completed an online survey designed to measure our four key study variables along with several other potentially relevant variables including sex, physical activity levels, and perceived COVID-19 impacts.

**Results:**

Over half of the participants reported moderate to extremely severe symptoms of anxiety and depression. Females reported stronger signs of SPS and AS and were more likely than males to have increased their moderate/vigorous exercise since the pandemic began. After controlling for the other variables, the best predictors of perceived COVID-19 impacts were SPS, childhood emotional abuse, and current levels of physical activity. Whereas all three personality variables and childhood emotional abuse emerged as significant predictors of both depression and anxiety, neither COVID-19 impacts nor physical activity levels accounted for unique variance in either model. Unexpectedly, male sex emerged as an additional risk factor for depression, raising the possibility that males experience unique stressors and societal pressures that increase their risk of depression.

**Discussion:**

These findings help to clarify the links between childhood emotional abuse, personality traits implicated in emotional awareness and self-regulation, and mental health. They may have important implications for the development and implementation of individualized treatments for common mental disorders.

## 1 Introduction

University students are at elevated risk for anxiety and depression (Kessler et al., 1994; Ibrahim et al., 2013; Li et al., 2022). Findings from a recent systematic review and meta-analysis by Sun et al. (2023) suggest that this situation has been made even worse since the coronavirus disease 2019 (COVID-19) was declared a pandemic by the World Health Organization (2020). Given this, it is particularly important to identify risk and protective factors for poor mental health in this vulnerable group.

Personality variables represent an important class of individual difference factors that relate to emotion generation and experience (Smith et al., 2018). Personality also interacts with situational context in predicting emotion regulation (Kobylińska and Kusev, 2019). This may explain why certain dimensions of personality have been found to predict both mental health and emotional responses to the pandemic (Osimo et al., 2021). In the present study, we investigated links between mental health and three personality traits that impact emotional awareness and self-regulatory processes, namely alexithymia, sensory processing sensitivity (SPS), and anxiety sensitivity (AS). All three are considered to be partially heritable (Stein et al., 1999; Jørgensen et al., 2007; Assary et al., 2021).

Sifneos (1973) described alexithymia as a trait marked by difficulties labeling one’s emotions and distinguishing them from bodily sensations, a lack of imaginative abilities, and a tendency to focus on the external environment at the expense of one’s inner thoughts and feelings. Individuals with alexithymia have also been found to experience high levels of emotional reactivity (Jakobson and Rigby, 2021), particularly when processing negative emotions (Preece et al., 2020). Key features of SPS include being unusually sensitive to subtle internally- and externally-generated stimuli, being easily overwhelmed or made uncomfortable by certain forms of sensory stimulation, having a rich inner life, and engaging in complex (“deep”) forms of cognitive processing (Aron et al., 2012). AS is characterized by a fear of anxiety-related physical sensations (Reiss et al., 1986) and the tendency to catastrophically misinterpret benign physiological sensations that often accompany anxiety (such as an increase in heart rate) as dangerous (McNally, 1999, 2002).

Given the overlap in features associated with alexithymia, SPS, and AS it is perhaps not surprising to find that they can co-occur (Devine et al., 1999; Rigby et al., 2020; Jakobson and Rigby, 2021) and that all three are related to adverse mental health outcomes (Taylor and Bagby, 2004; Liss et al., 2005; Deacon and Abramowitz, 2006). Despite this, they are rarely studied together, making it difficult to assess the role that each might play in determining mental health outcomes, or to ascertain how they relate to a range of other proposed risk and protective factors including, importantly, exposure to emotional abuse or other adverse experiences in childhood. Our primary aim was to tease apart the variance in university students’ mental health outcomes that is uniquely attributable to each of these variables.

In the following sections we review some of the relevant background literature in this area. As our research was conducted during the COVID-19 pandemic, we begin by examining risk and protective factors that could be exacerbating university students’ mental health concerns during this stressful time, including how the pandemic may have impacted engagement in physical activity levels and exercise routines. Following this, we discuss recent research examining links between the personality variables of interest, exercise, emotional abuse experienced in childhood, and mental health. Throughout the literature review we also consider possible sex differences in our study variables; this is important because females are considered to be at higher risk of experiencing symptoms of anxiety and depression than males, and this situation appears to have gotten worse during the current pandemic (Sun et al., 2023).

### 1.1 Risk and protective factors related to the pandemic and to engagement in physical activity

There are undoubtedly a number of pandemic-related factors that could be exacerbating mental health concerns in university students. For instance, disruptions due to the pandemic and doubts related to the federal government’s ability to respond to it have been shown to predict depressive symptoms in U.S. college students (Stamatis et al., 2022). The finding that females are at higher risk than males for mental health concerns (Sun et al., 2023) may relate, in part, to the fact that males and females have responded to the pandemic differently. For instance, males have been found to be less likely to follow pandemic related restrictions and guidelines (Litton, 2020), which could explain their higher COVID-19 mortality (Reeves and Ford, 2020). Conversely, females have reported increased levels of pandemic-related stress (Yan et al., 2021) and distress (Liu et al., 2020); this may be partly due to occupying a greater percentage of jobs that were heavily impacted by COVID-19, such as those in retail and healthcare (Xiong et al., 2020), and partly to increased demands of childcare (Pierce et al., 2020).

Like other members of the general population, university students have faced a range of additional challenges during the pandemic that may have negatively impacted their mental health, such as changes in access to medical and mental health care, and increased stress and discord in the family. The onset of the pandemic also resulted in many restrictions being put into place to try to reduce the spread of the virus, including social distancing and social isolation. This has likely contributed to increased feelings of loneliness—a factor that has emerged as an important predictor of mental health difficulties in young adults (Lee et al., 2020). However, the closure of recreation facilities and gyms also made engaging in physical activity more challenging for some (Wright et al., 2021). This is significant as a systematic review by Wolf et al. (2021) suggested that those who engaged in frequent exercise during the pandemic reported fewer symptoms of depression and anxiety than those who did not. This finding is not surprising, given that the benefits of physical activity for physical and mental health are well documented in the literature (Wahid et al., 2016; Pearce et al., 2022).

In addition to considering current activity levels, it is important to consider how, or if, an individual may have altered their exercise routine after the pandemic began, as these alterations could, themselves, be important predictors of mental health. Research suggests that, in general, levels of physical activity fell after the onset of the pandemic (Caputo and Reichert, 2020). Both the abrupt cessation of physical activity and increases in sedentary activities have been tied to increased depression and anxiety, and reduced quality of life (Weinstein et al., 2017; Runacres et al., 2021). In contrast, maintaining one’s pre-pandemic levels of physical activity appears to have been beneficial (Meyer et al., 2020). Interestingly, results from a study of 1,098 Canadians found that those who were less active during the pandemic had generally reduced their activity since its onset, whereas those who were more active had generally increased their activity (Lesser and Nienhuis, 2020). This is consistent with earlier findings suggesting that, during stressful times, inactive individuals decrease their activity while active individuals increase their activity, possibly reflecting different coping strategies (Stults-Kolehmainen and Sinha, 2014). Lesser and Nienhuis (2020) found that inactive (but not active) individuals who decreased activity reported worse psychological health and more signs of generalized anxiety than those who maintained or increased activity. Thus, at least in those who are not particularly active, maintaining or increasing physical activity seems to have been protective.

### 1.2 Alexithymia

Alexithymia is considered a vulnerability factor for developing depression and anxiety (Taylor and Bagby, 2004). Moreover, in those who exercise frequently it may also increase risk for a form of exercise dependence that is characterized by severe depression (Van Landeghem et al., 2019). Recent research shows that individuals reporting higher levels of alexithymia have had worse emotional responses to the pandemic (Osimo et al., 2021) and greater health anxiety (Gürsoy et al., 2021) compared to those reporting lower levels of this trait. Interestingly, Tang et al. (2020) suggested that alexithymia mediates the link between exposure to the COVID-19 pandemic (including direct or indirect exposure to the virus itself or to stressful media messages) and symptoms of PTSD and depression among university students. This makes sense, as individuals who have trouble identifying and labeling their emotions are also likely to struggle with using reappraisal to regulate their emotions (Connelly and Denney, 2007; van der Meer et al., 2009), thereby increasing risk for psychopathologies characterized by emotion dysregulation (Preece et al., 2017).

It is unclear whether sex differences in alexithymia may have contributed to sex differences that have been observed in levels of pandemic-related stress/distress (Liu et al., 2020; Yan et al., 2021) or mental health (Sun et al., 2023). Indeed, the evidence for sex differences in alexithymia itself is mixed. Levant et al. (2009) argued that males experience higher mean levels of alexithymia than females due to the traditional socialization of males to show a more restricted range of emotions than females (the normative male alexithymia hypothesis). However, sex had a small effect size (Hedges’ *d* = 0.22) in this meta-analysis, which involved primarily clinical samples. In a review of 32 studies conducted in non-clinical populations, 17 found that males reported higher mean levels of alexithymia than females, one found that females reported higher mean levels of alexithymia than males, and 14 found no significant gender differences (Levant et al., 2006). Despite the mixed findings, it has been recommended (Campanella et al., 2012) that individual difference factors such as alexithymia should be considered when examining sex differences in emotional processing.

### 1.3 Sensory processing sensitivity

SPS is a risk factor for anxiety and depression (Aron et al., 2012; Jakobson and Rigby, 2021). Individuals with SPS often unconsciously withdraw from society due to feeling over-aroused and over-stimulated (Aron and Aron, 1997), and this could reduce participation in exercise/sport. Research conducted during the pandemic suggests that SPS is also a predictor of increased pandemic related stress in older adolescents and young adults (Iimura, 2022) and of increased levels of health anxiety in adults (Güneş and Bulut, 2022). In adolescents, perceived COVID-19 impact was found to partially mediate the link between SPS and internalizing symptoms; thus, those displaying stronger signs of SPS reported being more negatively impacted by the pandemic and this, in turn, predicted stronger internalizing symptoms (Burgard et al., 2022). Interestingly, however, results from another study suggested that the negative effects of SPS on pandemic related stress could be mitigated by resilience, suggesting that resilience is an important protective factor in individuals with high levels of this trait (Iimura, 2022). Women have been found to report higher levels of SPS than men (Aron and Aron, 1997; Benham, 2006); however, some researchers have suggested that this may be partly due to Western cultural norms, where sensitivity is seen as being less acceptable in males (Aron and Aron, 1997).

### 1.4 Anxiety sensitivity

AS is associated with many emotional disorders, including generalized anxiety disorder (Deacon and Abramowitz, 2006) and depression (Carleton et al., 2009). Interestingly, although individuals with AS are generally motivated to avoid exercise (Sabourin et al., 2011; Moshier et al., 2015; Farris et al., 2019), exercise can be effective in reducing AS (Broman-Fulks and Storey, 2008; Watt et al., 2008; Sabourin et al., 2015) leading to improvements in mental health (Broman-Fulks et al., 2018). Research suggests that females score higher than males on AS (Stewart et al., 1997). Moreover, AS has been found to be a mediator between sex and pandemic-related distress (i.e., news anxiety and overall anxiety), suggesting that higher levels of AS might help explain increased distress related to the pandemic in females, specifically (DeGrace et al., 2021).

### 1.5 Emotional abuse and other forms of adversity experienced in childhood

It is important to consider the role that adverse childhood experiences play in predicting mental health. In one of the earliest and most influential papers on the subject, Felitti et al. (1998) characterized adverse childhood experiences as falling into seven areas: three were associated with childhood abuse (i.e., psychological abuse, physical abuse, sexual abuse) and four with aspects of household dysfunction (i.e., substance abuse, mental illness, violent treatment of mother or stepmother, and criminal behavior). These authors reported a strong, graded relationship between the number of adverse childhood experiences one had been exposed to and experiences of physical and mental illness in adulthood. Interestingly, exposure to such experiences has also been shown to reduce participation in sport (Noel-London et al., 2021). This latter finding is particularly unfortunate, as engagement in exercise/sport is believed to be an important protective factor for those who have experienced childhood adversity (Norris and Norris, 2021).

Research into the frequency and impact of adverse childhood experiences on emotion regulation and both anxiety-related and mood disorders has proliferated. For example, data from the 2012 Canadian Community Health Survey: Mental Health suggested that the prevalence of child abuse (including physical abuse, sexual abuse, or exposure to intimate partner violence) was 32%, and that exposure to such abuse was associated with an increased risk of depression, bipolar disorder, generalized anxiety disorder, and alcohol and drug abuse/dependence (Afifi et al., 2014). The focus of the present study was on emotional abuse experienced in childhood and adolescence. Based on their review of meta-analytic studies, Stoltenborgh et al. (2015) found a global prevalence rate from self-report studies of 36.3% for this type of abuse—a rate much higher than that seen for sexual abuse, which has received much more attention from researchers. It is important to expand our understanding of the potential impacts of emotional abuse, which has been found to be associated with chronic problems with anxiety and/or depression that persistent into adulthood (Hovens et al., 2012). Research examining possible sex differences in exposure to this form of abuse are mixed, with some studies reporting higher rates in females (Scher et al., 2004; Mathews et al., 2023), others reporting higher rates in males (Simsek et al., 2017), and others finding no differences between sexes (Akgöz Aktaş et al., 2023).

A recent longitudinal study that spanned the first year of the COVID-19 pandemic highlighted the important role that childhood emotional abuse played in increasing risk for poor mental health in third-year medical students (Stanislawski et al., 2023). Nearly three-quarters of the student sample screened positive for depression, anxiety, or PTSD over the course of the study. Despite the vast majority of the students having experienced trauma and/or pandemic-related stressors during their clerkships, pandemic-related worries decreased over time and did not predict depression, anxiety, and/or PTSD at year end, whereas baseline psychological distress, resilience, and childhood emotional abuse did. Conducting further research examining how personality variables may interact with these variables may provide important insights into the mechanisms underlying these associations, and targets for intervention.

Individuals who were exposed to childhood abuse report higher levels of alexithymia (Khan and Jaffee, 2022) and Karaca Dinç et al. (2021) presented evidence that alexithymia mediates the relationship between childhood trauma and psychopathology, including depression, anxiety, and negative self-esteem. Interestingly, these authors also found that the relationship between childhood trauma and increased negative affectivity was mediated by SPS. The latter finding is consistent with the fact that highly sensitive individuals are more likely to experience anxiety and depression (Aron et al., 2005), neuroticism (Aron et al., 2012), emotion dysregulation, and psychopathology (Greven et al., 2019) if they were raised in a negative childhood environment or exposed to trauma. In contrast, when individuals with SPS are raised in a supportive environment they flourish, perhaps because they learn how to process and regulate responses to negatively valenced stimuli and find positively valenced stimuli highly rewarding (Acevedo et al., 2017).

Research has also explored links between childhood adversity and AS. Studies in children suggest that stressful life experiences (i.e., health-related concerns and family discord; McLaughlin and Hatzenbuehler, 2009) and experiences of abuse and neglect (Martin et al., 2014) are positive predictors of AS. AS has also been shown to mediate the relationships between childhood abuse and substance use relapse risk (King et al., 2020), stressful life events and anxiety (McLaughlin and Hatzenbuehler, 2009), and childhood emotional trauma and internalizing symptoms related to anxiety and depression (Yang et al., 2022).

### 1.6 The current study

The preceding discussion highlights the fact that there are strong links between the risk and protective factors for poor mental health that are the focus of the present investigation. The first key objective of the present study was simply to present data collected during the COVID-19 pandemic on levels of alexithymia, SPS, AS, childhood emotional abuse, pandemic-related impacts, anxiety, and depression in a large, mixed-sex sample of university students. Although this was done largely for descriptive purposes (to characterize our sample), based on the literature reviewed above we expected rates of anxiety and depression to be high, and that many of the study variables listed above would be positively related with one another.

The second objective was to test for possible sex differences in our study variables. This was of interest given mixed findings that have been reported for some of these variables in the literature. Here, it was expected that females would score higher than males on measures of SPS, AS, pandemic-related impacts, anxiety, and depression. It was unclear if the two groups would differ on alexithymia or on experiences of childhood emotional abuse.

The third objective was to examine how the pandemic may have differentially impacted male and female students’ exercise routines and whether this was related to their current mental health. Addressing this question adds to the literature exploring links between physical activity and psychological well-being. It was expected that decreasing one’s activity after the onset of the pandemic would be a risk factor for poor mental health in both sexes, whereas maintaining or increasing physical activity would be protective.

Finally, the fourth objective was to assess the unique contributions that personality variables and childhood emotional abuse made to the prediction of mental health when controlling for current levels of physical activity, COVID-19 impacts, and sex. Based on the literature reviewed above, one might expect to find that scoring high on the personality variables, reporting more childhood emotional abuse and COVID-19 impacts, and being female would all increase risk for poor mental health, while engaging in more physical activity would be a protective factor. As noted earlier, however, these variables are frequently confounded. By teasing apart the unique contribution that each of our study variables made to the prediction of mental health it was hoped that a clearer understanding of risk and protective factors impacting university students during this stressful time would be gained. Ultimately, the findings may have important implications for theory and clinical practice.

## 2 Materials and methods

### 2.1 Participants

The majority of participants were recruited using convenience sampling from the Introduction to Psychology subject pool at the University of Manitoba. In an effort to increase involvement of athletes, materials were also distributed to members of university varsity sports teams, but only four additional participants were recruited through this route. Data were collected between February 11 and March 26, 2021, which coincided with a period between the first and second waves of the COVID-19 pandemic in Manitoba, when case counts were generally less than 100 per day (Dong et al., 2020).

Details regarding data cleaning are provided below. The final sample included 410 individuals with a mean age of 19.7 years (*SD* = 3.4; range 17–44). The majority identified as female (75.4%) and were Canadian citizens (83.7%). Although a large proportion identified as White/European (44.7%), almost one third of the sample (31.2%) identified as Filipino or Asian/Southeast Asian, 7.8% identified as Black, and 5% identified as First Nation/Métis.

### 2.2 Procedures

Due to pandemic-related restrictions on in-person testing, participants completed an on-line survey that included measures tapping into demographics, personality variables (alexithymia, SPS, and AS), emotional abuse experienced in childhood, current levels of physical activity, the impact of the current pandemic in multiple domains including changes to their exercise routines, and current levels of depression and anxiety. We also included a measure of conscientious responding to check for poor effort. Data were also gathered on several other variables for a separate investigation. The survey was administered via the Qualtrics platform and participants completed it remotely at a time and location of their choosing. All procedures were approved by the Psychology/Sociology Research Ethics Board at the University of Manitoba. Participants gave informed consent and were compensated by receiving credit toward a research participation option in their course, or a small honorarium. Records were maintained in compliance with the Personal Health Information Act.

### 2.3 Measures

#### 2.3.1 Demographics

Participants reported on their age, sex, race, and citizenship.

#### 2.3.2 Toronto Alexithymia Scale-20

The Toronto Alexithymia Scale-20 (TAS-20; Bagby et al., 1994) is a widely used instrument designed to measure alexithymic traits. Participants rated how well each of 20 items described them using a 5-point Likert scale ranging from 1 (*Strongly disagree*) to 5 (*Strongly agree*). Items belong to one of three subscales which tap into key features of alexithymia: (1) difficulty identifying feelings (7 items; e.g., “I have feelings that I cannot quite identify”); (2) difficulty describing feelings (5 items; e.g., “It is difficult for me to find the right words about my feelings”); and (3) externally-oriented thinking (8 items; e.g., “I prefer to analyze problems rather than just describe them”). After reverse-scoring select items, ratings were summed to create a total score that could range from 20 to 100. Traditionally, total scores ≥61 are said to signify alexithymia, scores ≤51 are said to signify lexithymia, and scores falling in between these cut points are classified as borderline (Parker et al., 2003); however, following recommendations by Bagby et al. (2020), we treated alexithymia as a continuous variable in our analyses.

Previous work confirms good internal consistency for total scores and subscale scores of the TAS-20 (Parker et al., 2003). Indeed, a recent review of over 25 years of psychometric literature on this measure suggests that researchers can be confident in its reliability and validity as a measure of alexithymia (Bagby et al., 2020). The internal consistency of the total score in the current study was good (α = 0.84).

#### 2.3.3 Highly Sensitive Person Scale

The Highly Sensitive Person Scale (HSPS; Aron and Aron, 1997) measures SPS. Participants rated how well each of 27 items described them using a 7-point Likert scale ranging from 1 (*Not at all*) to 7 (*Extremely*). A total score out of seven was obtained by computing the mean rating across all items. Recent work from Lionetti et al. (2018) suggests that individuals can be grouped into low, moderate, and high levels of SPS by using cut-scores corresponding to the 30th and 70th percentiles. These groups are labelled *dandelions*, *tulips*, and *orchids*, respectively. Dandelions do well in most circumstances, whereas orchids are highly sensitive and require ideal early environments to thrive. Tulips fall somewhere between these two extremes.

Confirmatory factor analysis has suggested that the HSPS taps into three facets of SPS: ease of excitation, low sensory threshold, and aesthetic sensitivity (Smolewska et al., 2006). The ease of excitation subscale (12 items) measures how strongly one is affected by bodily cues (e.g., pain, hunger) and how well one manages in a busy sensory environment and when multitasking (e.g., “Does being very hungry create a strong reaction in you, disrupting your concentration or mood?”). The low sensory threshold subscale (6 items) measures how strongly one is negatively impacted by sensory experiences and avoids them (e.g., “Do you make it a high priority to arrange your life to avoid upsetting or overwhelming situations?”). Finally, the aesthetic sensitivity subscale (7 items) measures aesthetic sensitivity (e.g., “Do you notice and enjoy delicate or fine scents, tastes, sounds, work of arts?”). Previous works confirm adequate to excellent internal consistency and validity of the total score and subscale scores of the HSPS (Smolewska et al., 2006; Evers et al., 2008; Liss et al., 2008; Ahadi and Basharpoor, 2010; Gerstenberg, 2012). Only total scores were used in the present investigation; the internal consistency of the total score in the current study was good (α = 0.88).

#### 2.3.4 Anxiety Sensitivity Index-3

The Anxiety Sensitivity Index (ASI-3; Taylor et al., 2007) measures AS. Participants rated how strongly they agreed or disagreed with each of 18 items using a 5-point Likert scale ranging from 0 (*very little*) to 4 (*very much*). Items belong to one of three, 6-item subscales that tap into three domains of concern: (1) physical (e.g., “It scares me when my heart beats rapidly”); (2) cognitive (e.g., “When my mind goes blank, I worry there is something terribly wrong with me”); and (3) social (e.g., “It scares me when I blush in front of people”). Only the total score, which is obtained by summing all ratings (possible range 0–72) was used in the present investigation. Higher total scores indicate greater symptom severity. Previous work confirms good internal consistency of the ASI-3 (Taylor et al., 2007). The internal consistency of the total score in the current study was excellent (α = 0.91).

#### 2.3.5 Emotional Abuse Subscale of the Childhood Trauma Questionnaire Short Form

The short form of the CTQ (Bernstein et al., 2003) measures five different forms of abuse experienced in childhood, including physical, sexual, and emotional abuse, and physical and emotional neglect. Only the 5-item emotional abuse subscale was used in the current study. A sample item is “When I was growing up people in my family said hurtful or insulting things to me.” Responses were made on a 5-point Likert scale ranging from 1 (*never true*) to 5 (*very often true*). Past work suggests excellent internal consistency of the CTQ (short form) in adolescent and community member samples (Bernstein et al., 2003). The internal consistency of the Emotional Abuse Subscale of the Childhood Trauma Questionnaire short form (EA-CTQ) used in the current study was good (α = 0.87).

#### 2.3.6 International Physical Activity Questionnaire – Short Form

The International Physical Activity Questionnaire (IPAQ) was developed by a group of experts between 1997 and 1998 to provide a global standard by which to measure physical activity (Craig et al., 2003). The 7-item short form measures total activity levels, as well as time spent sitting. We used the version in which participants are asked to report how many days, and how much time per day, they engage in vigorous activity, moderate activity, walking, and sitting during a “usual week.” In the present study, we modified this measure by adding four items to gather information about whether the time participants spent in each of these four types of activities had increased, decreased, or not changed since the onset of the pandemic, bringing the total number of items to 11.

The IPAQ uses multiples of the resting metabolic rate (MET) units to quantify physical activity. A MET-minute for walking or engaging in either moderate or vigorous activity is calculated by multiplying the MET score for that activity (walking = 3.3; moderate activity = 4.0; vigorous activity = 8.0) by the amount of time (in minutes) it was performed. This score is then multiplied by the number of days per week the activity was engaged in. A total MET-minutes/week (Total MET) score is found by summing the scores across the three types of activities. In addition to extracting this continuous measure, participants can be grouped into three categories based on activity level (low, moderate, and vigorous) using criteria outlined in the standard scoring procedures.

In general, physical activity questionnaires lack rigorous methodological testing (van Poppel et al., 2010). Of the available options, the IPAQ short form is recommended for research (van Poppel et al., 2010), as previous work confirms good test–retest reliability and construct validity has been established by comparing the IPAQ short form to objective measures of exercise, such as those obtained through use of an accelerometer (Craig et al., 2003). Importantly, reliability is similar for the version of the IPAQ in which participants reflect on the “last 7 days” and the version used in the present study where they reflect on a “usual week” (Craig et al., 2003).

#### 2.3.7 Modified Coronavirus Impact Scale (mCIS)

The original Coronavirus Impact Scale (CIS; Stoddard et al., 2023) is a brief, self-report tool designed to measure the extent to which the current pandemic has impacted participants’ lives in the following eight areas: routines, family income/employment, food access, medical health care access, mental health treatment access, access to extended family and non-family social supports, experiences of stress related to the coronavirus pandemic, and stress and discord in the family. In the present study, the scale was modified by adding one item to assess the impact of COVID-19 on stress related to changes in physical activity/participation in sport, specifically. Participants indicated their responses to each of the nine items using a 4-point Likert scale ranging from 0 (*None*) to 3 (*Severe*), and scores were summed to yield a total COVID-19 impact score. The internal consistency of the mCIS was 0.75, which is considered to be acceptable (van Griethuijsen et al., 2014; Taber, 2018).

In addition to the above, the original CIS includes three items that ask respondents to indicate if they, an immediate family member, or an extended family member or friend had contracted coronavirus and, if so, to rate the severity of illness. Responses to these items are not reflected in the total score.

#### 2.3.8 Depression and Anxiety Subscales of the Depression Anxiety Stress Scale (DASS-21)

The DASS-21 (Lovibond and Lovibond, 1995) measures symptoms of depression, anxiety, and stress; only the anxiety and depression subscales were administered in the current investigation. Participants rated the extent to which each of 14 statements applied to them over the past week using a 4-point Likert scale ranging from 0 (*Did not apply to me at all – NEVER*) to 3 (*Applied to me very much, or most of the time – ALMOST ALWAYS*). A total score out of 42 was obtained for each of the two subscales by summing ratings of relevant items and then multiplying by two, with higher scores indicating greater severity of symptoms. Individuals can be assigned to one of five categories of symptom severity (*normal, mild*, *moderate*, *severe*, and *extremely severe*) based on cut points for depression and anxiety provided in the manual. Previous work confirms good internal consistency of the DASS-21 and good concurrent validity with the Beck Depression Inventory (Antony et al., 1998). In the present study, the internal consistency of the depression (α = 0.90) and anxiety (α = 0.83) subscales of the DASS-21 were good.

#### 2.3.9 Attention checks: Conscientious Responders Scale

The 5-item Conscientious Responders Scale measures conscientious (i.e., honest and accurate) responding (Marjanovic et al., 2014). It can be used to detect poor effort in individuals completing self-report measures and increase the validity of survey research. Each item has a correct answer (e.g., “To answer this question, please choose option number four, “neither agree nor disagree”), which allows one to determine whether particular instructions are being followed or not. This measure has been shown to distinguish conscientious from random responding with greater than 93% accuracy (Marjanovic et al., 2014). The five items were randomly distributed throughout all other measures.

### 2.4 Data cleaning

There were 511 respondents. They were asked to identify themselves by name and email address to increase the validity of their responses (Meade and Craig, 2012). One respondent did not provide informed consent and was, therefore, excluded. A number of additional exclusions were made to improve data quality. First, duplicate entries were removed (*n* = 2); in these cases, only the first entry was retained. Second, participants who failed to complete at least one subscale of any given questionnaire (*n* = 4) were excluded. Third, participants who scored at or above the 99^th^ percentile on time-to-completion were excluded (*n* = 4). Fourth, following recommendations by Marjanovic et al. (2014), participants who failed to correctly answer three or more items on the Conscientious Responders Scale were excluded (*n* = 12).

In addition to the above exclusions, participants who failed to properly complete a questionnaire or provided questionable/ambiguous responses were excluded (*n* = 78). In all but three cases, these exclusions were based on responses on the IPAQ. The 75 individuals excluded based on IPAQ responses (a) had missing data, (b) had Total MET scores that were outliers, and/or (c) reported not exercising at a particular level (vigorous, moderate, walking) despite indicating that they had *increased* exercising at that same level since the onset of the pandemic. We acknowledge that imposing the latter (non-standard) criterion may have led us to exclude some individuals who had briefly increased their activity level but then tapered off. Nonetheless, our goal was to try to include only those individuals who reported an increase in exercise at a given level and were able to sustain it to some degree. That these exclusions did not create a biased sample is supported by the observation that the demographics of the group of individuals who were excluded were very similar to the demographics of those included in the final sample (see Participants). Thus, the majority of excluded individuals were female (78.6%) and Canadian citizens (76.8%). Their mean age was 20.1 years (*SD* = 3.0; range 17–30) and the majority identified as White/European (44.6%) or as Filipino or Asian/Southeast Asian (41.1%).

In the final sample of 410 individuals there were between 0.0 and 0.4% missing data per scale/subscale. Using Little’s MCAR test, data were found to be missing completely at random, *p* ≥ 0.08. Expectation maximization was used to estimate missing data values. Here and below, all statistical analyses were performed using SPSS (v. 28) software.

## 3 Results

### 3.1 Objective 1: Characterizing the full sample

**Figure 1:**
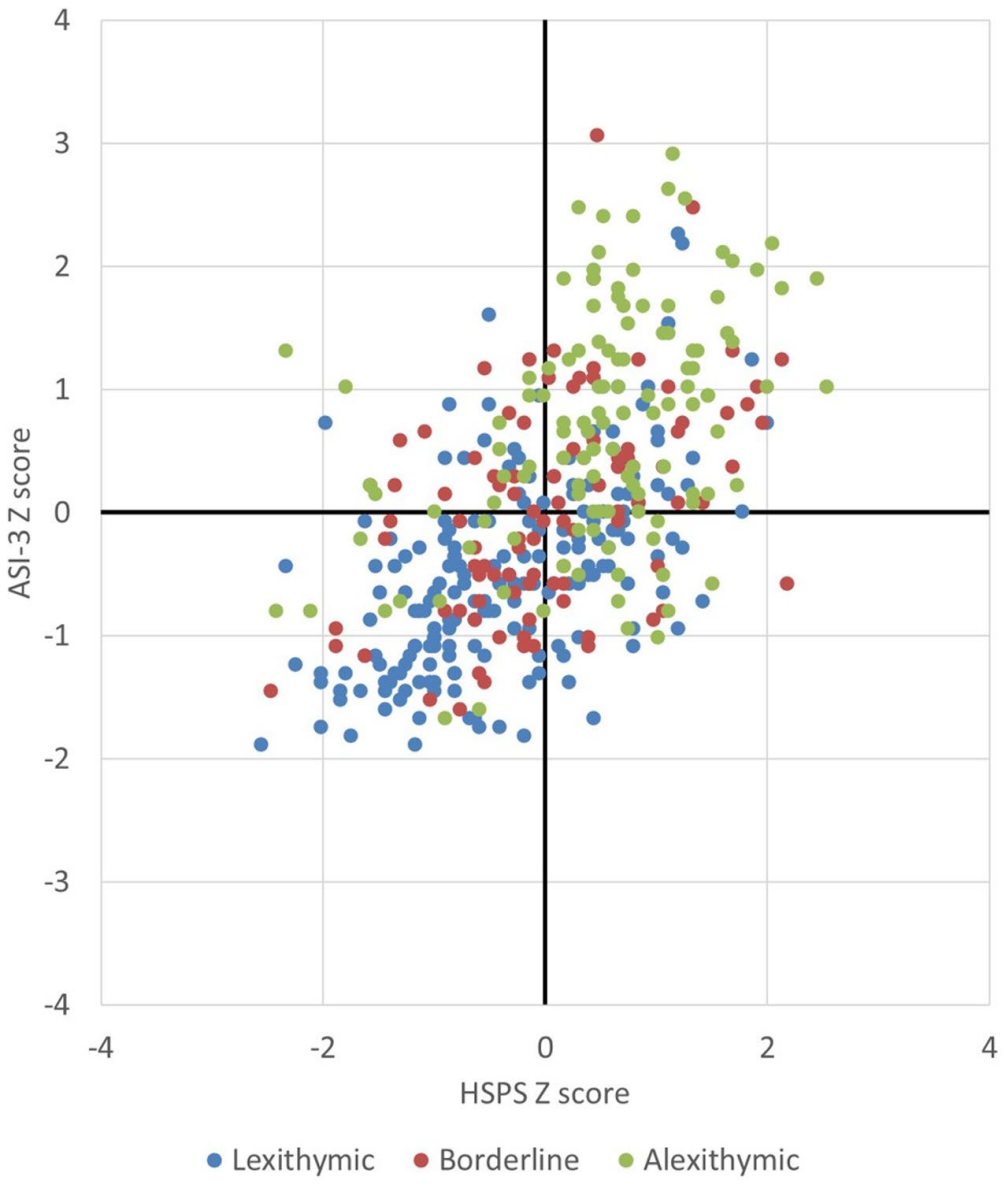
Shown is the distribution of scores on the Highly Sensitive Person Scale and the Anxiety Sensitivity Index-3 (expressed as z-scores) in individuals scoring in the lexithymic, borderline, and alexithymic ranges on the 20-item Toronto Alexithymia Scale.

A first key objective of the current work was to characterize the full sample of 410 individuals by providing descriptive statistics on the study variables. This was done to facilitate comparisons with samples drawn in other studies involving university students engaged in varying levels of exercise/sport, and to illustrate the correlations between the study variables. In the full sample, scores on all variables covered virtually the whole range (see Table 1). Most variables were positively correlated with one another, with moderate to large effect size (see Table 2). Almost one-third of the sample (30.5%) had TAS-20 total scores that fell at or above the traditional cut-off for alexithymia (i.e., ≥61). As can be seen in Figure 1, whereas the majority of these individuals (66.4%) had above-average scores on both the HSPS and the ASI-3, the majority (57.4%) of individuals scoring in the lexithymic range (i.e., ≤51) had below-average scores on both of these measures, *Χ*^2^(6) = 101.3, *p* < 0.001. This speaks to the strong associations between these three traits. Based on their responses to the DASS-21, over half of the sample reported moderate to extremely severe symptoms of depression (52.9%) and anxiety (52.9%).

**Table 1:** Descriptive statistics for study variables (*N* = 410).

	*M*	*SD*	Minimum	Maximum
TAS-20	53.1	11.2	24	83
HSPS	4.3	0.8	2.2	6.4
ASI-3	25.9	13.7	0	68
EA-CTQ	10.1	5.2	5	25
mCIS	8.8	3.6	0	18
DASS depression	15.3	10.8	0	42
DASS anxiety	11.9	9.0	0	40

	Mdn	Interquartile range
Total METs	2,190	2,910

TAS-20, Toronto Alexithymia Scale; HSPS, Highly Sensitive Person Scale; ASI-3, Anxiety Sensitivity Index; EA-CTQ, Emotional Abuse Subscale of the Childhood Trauma Questionnaire short form; mCIS, Coronavirus Impact Scale; DASS Depression and DASS Anxiety, Depression and Anxiety Subscales of the 21-Item Depression Anxiety Stress Scale; Total METs, measure of overall weekly activity.

**Table 2:** Bivariate correlations between study variables (*N* = 410).

	2^a^	3^a^	4^a^	5^a^	6^a^	7^a^	8^b^	9^b^
1 TAS-20	0.39^***^	0.54^***^	0.31^***^	0.27^***^	0.55^***^	0.49^***^	−0.06	−0.07
2 HSPS	–	0.58^***^	0.36^***^	0.33^***^	0.42^***^	0.48^***^	−0.10^*^	−0.12^*^
3 ASI-3		–	0.43^***^	0.33^***^	0.55^***^	0.68^***^	0.01	−0.01
4 EA-CTQ			–	0.34^***^	0.48^***^	0.44^***^	−0.02	−0.01
5 mCIS				–	0.29^***^	0.32^***^	0.17^***^	0.15^**^
6 DASS D					–	0.61^***^	0.00	−0.02
7 DASS A						–	0.01	−0.04
8 Total METs							–	0.86^***^
9 IPAQ Code								–

TAS-20, Toronto Alexithymia Scale; HSPS, Highly Sensitive Person Scale; ASI-3, Anxiety Sensitivity Index; EA-CTQ, Emotional Abuse Subscale of the Childhood Trauma Questionnaire short form; mCIS, Coronavirus Impact Scale; DASS D and DASS A, Depression and Anxiety Subscales of the 21-Item Depression Anxiety Stress Scale; Total METs, measure of overall weekly activity; International Physical Activity Questionnaire (IPAQ) Code: 1 = low, 2 = moderate, 3 = high.

^a^Pearson correlation.

^b^Spearman correlation.

^*^p < 0.05, ^**^p < 0.01, ^***^p < 0.001 (2-tailed).

The majority of the sample (78.2%) were classified as moderately or highly active. The Total METs score (which provided a measure of overall weekly activity) and the IPAQ code (which was used to group participants according to whether they engaged in low, moderate, or high levels of activity) were strongly positively correlated with one another. They were also negatively correlated with HSPS scores and positively correlated with mCIS scores (with small effect size) but, contrary to expectations, they were not correlated with either depression or anxiety.

### 3.2 Objective 2: Testing for possible sex differences

A second key objective of the present research was to examine possible sex differences in the study variables. Four individuals either did not disclose their sex or did not identify as either male or female; these individuals were excluded from the analyses, and those addressing the third and fourth study objectives (see below). This left samples of 97 males (*M*_age_ = 20.5 years, *SD* = 4.5) and 309 females (*M*_age_ = 19.5 years, *SD* = 3.0).

A series of Welch’s t-tests was conducted to look for possible sex differences in the study variables (see Table 3). The Bonferroni correction for multiple tests was used, and a comparison was considered significant if *p* ≤ 0.007. Results indicated that there was no sex difference in mean alexithymia scores and a follow-up chi-square test confirmed that the proportion of individuals who scored above the traditional cut-off for alexithymia was similar in males and females. However, as expected, females had higher mean scores than males on the HSPS, and a follow-up chi-square test confirmed that there was a significant sex difference in the distribution of dandelions, tulips, and orchids [*Χ^2^*(2) = 24.031, *p* < 0.001], with more than twice as many females as males being classified as highly sensitive (35.0% vs. 15.5%) based on the criterion of scoring in the upper 30^th^ percentile of the distribution of HSPS total scores in the full sample. In addition to the above, as predicted, females also scored significantly higher than males on the ASI-3.

**Table 3:** Similarities and differences between males and females.

	Males (*n* = 97)		Females (*n* = 309)			
	*M*	*SD*	*M*	*SD*	*t*	*p*
TAS-20	50.9	11.1	53.6	11.1	4.65	0.033
HSPS	4	0.8	4.5	0.8	30.3	<0.001
ASI-3	22.2	13.8	26.8	13.5	8.51	0.004
EA-CTQ	9.2	5.1	10.4	5.1	3.53	0.062
mCIS	7.8	3.7	9.1	3.5	9.28	0.003
DASS D	14.6	10.9	15.3	10.7	0.31	0.579
DASS A	10.5	8.1	12.3	9.2	3.42	0.066
Total METs	2,875	2,293	2,553	2,137	1.51	0.221

TAS-20, Toronto Alexithymia Scale; HSPS, Highly Sensitive Person Scale; ASI-3, Anxiety Sensitivity Index; EA-CTQ, Emotional Abuse Subscale of the Childhood Trauma Questionnaire short form; mCIS, Coronavirus Impact Scale; DASS D and DASS A, Depression and Anxiety Subscales of the 21-Item Depression Anxiety Stress Scale; Total METs, measure of overall weekly activity.

The sexes did not differ in their current, weekly activity levels (Total METs), and a chi-square test confirmed that the proportions of males and females exercising at low, moderate, and high levels were similar [*Χ^2^*(2) = 4.96, *p* = 0.084]. Contrary to expectations, they also did not differ in self-reported depression, although there was a trend for females to report greater anxiety (see Table 3). A follow-up chi-square test revealed a trend for females to be less likely to score in the mild range and more likely to score in the very severe range for anxiety compared to males [*Χ^2^*(4) = 7.93, *p* = 0.094].

As predicted, females scored higher on the mCIS (see Table 3). To determine whether the groups differed in any of the nine specific domains sampled with this measure, chi-square tests were once again utilised. We had no predictions regarding these specific variables; the results are presented here for descriptive purposes only. A Bonferroni correction for multiple tests was used, and an alpha level of 0.006 was adopted for all comparisons. There were no significant differences between groups on negative impacts related to changes in routines, family income, stress in the family, food access, access to mental or medical health care, or stress associated with changes in exercise/participation in sport (as assessed by the item we added to the mCIS). However, females reported more moderate impacts than males related to stress due to the pandemic itself and to changes in social supports [*Χ^2^*(2) ≥ 10.172, *p* ≤ 0.006 in both cases].

Given that females scored higher than males on the HSPS and AS and showed a tendency to be more anxious, overall, we ran an exploratory follow-up hierarchical regression to determine if these variables might account for the fact that females felt more negatively impacted by the pandemic than males. Sex and TAS-20, HSPS, ASI-3, EA-CTQ and physical activity levels (low, moderate, high) were entered at step 1, and anxiety and depression scores were entered at step 2. This allowed us to assess the impacts of HSPS, AS, and anxiety on the perceived impact of COVID-19 while holding other potentially relevant variables constant. The assumptions for multiple regression were met. The model was significant at both step 1 [*R*^2^ = 0.209, *F*(6, 399) = 17.62, *p* < 0.001] and step 2 [*R*^2^ = 0.214, *F*(8, 397) = 13.52, *p* < 0.001], however adding the mental health indicators at step 2 did not improve model fit significantly (*R*^2^ change = 0.005, *p* = 0.308). There was a trend for female sex to continue to predict higher mCIS scores in both models, but SPS, reporting a history of childhood emotional abuse, and engaging in higher levels of physical activity emerged as the strongest predictors when controlling for all other variables (see Table 4).

**Table 4:** Hierarchical regression predicting negative COVID-19 impacts (mCIS).

		*b*	*SE*	*β*	*p*
Step 1	(Constant)	−0.244	1.244		0.845
	Sex	0.677	0.391	0.080	0.084
	TAS-20	0.034	0.017	0.104	0.052
	HSPS	0.658	0.253	0.150	0.010
	ASI-3	0.025	0.016	0.094	0.134
	EA-CTQ	0.136	0.035	0.193	<0.001
	IPAQ Code	0.804	0.202	0.179	<0.001
Step 2	(Constant)	0.246	1.294		0.849
	Sex	0.729	0.393	0.086	0.064
	TAS-20	0.027	0.018	0.083	0.144
	HSPS	0.601	0.256	0.137	0.019
	ASI-3	0.013	0.018	0.048	0.483
	EA-CTQ	0.121	0.037	0.172	0.001
	IPAQ Code	0.799	0.202	0.178	<0.001
	DASS D	0.010	0.021	0.029	0.649
	DASS A	0.034	0.027	0.084	0.206

mCIS, modified Coronavirus Impact Scale; sex code: 0 = male, 1 = female; TAS-20, Toronto Alexithymia Scale; HSPS, Highly Sensitive Person Scale; ASI-3, Anxiety Sensitivity Index; EA-CTQ, Emotional Abuse Subscale of the Childhood Trauma Questionnaire short form; International Physical Activity Questionnaire (IPAQ) Code: 1 = low, 2 = moderate, 3 = high. Depression (DASS D) and anxiety (DASS A) measured using the Depression and Anxiety Subscales of the 21-Item Depression Anxiety Stress Scale.

### 3.3 Objective 3: Assessing sex differences relating to pandemic-related changes in physical activity and their links to mental health

Chi-square tests were used to assess possible sex differences in changes in activity since the onset of the pandemic. As expected, virtually all participants reported having increased the time they spent sitting; this was true of both males (91%) and females (96%; group difference not significant). There was no significant difference in the proportion of males and females who reported having not changed their physical activity levels (15% vs. 20%), or who reported having increased their walking (26% vs. 29%). However, females were more likely than males to report having increased their moderate exercise [22% vs. 9%, *Χ^2^*(2) = 8.335, *p* = 0.015] and their vigorous exercise [18% vs. 8%; *Χ^2^*(2) = 6.018, *p* = 0.049] since the onset of the pandemic. Contrary to expectations, whether participants of either sex had increased, decreased, or not changed their activity levels (vigorous/moderate exercise or walking) after the onset of the pandemic did not relate to anxiety or depression levels (Kruskal-Wallis tests, *p* ≥ 0.351 in each case).

### 3.4 Objective 4: Variables accounting for unique variance in current mental health

To identify variables that accounted for unique variance in the severity of symptoms of anxiety and depression, we conducted separate multiple regressions predicting depression and anxiety. TAS-20, HSPS, ASI-3, EA-CTQ, mCIS, physical activity levels (low, moderate, high), and sex were entered as predictors. The assumptions for multiple regression were met. The results are presented in Table 5.

**Table 5:** Predictors of depression and anxiety in university students.

	Mental health outcome							
Predictors	Depression				Anxiety			
	*b*	*SE*	*β*	*p*	*b*	SE	*β*	*p*
(Constant)	−15.346	3.094		<0.001	−10.129	2.446		<0.001
TAS-20	0.299	0.043	0.311	<0.001	0.110	0.034	0.136	0.001
HSPS	1.367	0.635	0.105	0.032	1.197	0.502	0.110	0.018
ASI-3	0.159	0.041	0.203	<0.001	0.303	0.032	0.461	<0.001
EA-CTQ	0.528	0.089	0.252	<0.001	0.261	0.070	0.149	<0.001
mCIS	0.108	0.124	0.036	0.384	0.145	0.098	0.058	0.143
IPAQ Code	0.043	0.512	0.003	0.933	0.001	0.405	0.000	0.998
Sex	−2.311	0.975	−0.092	0.018	−1.008	0.771	−0.048	0.192

TAS-20, Toronto Alexithymia Scale; HSPS, Highly Sensitive Persons Scale; ASI-3, Anxiety Sensitivity Index; EA-CTQ, Emotional Abuse Subscale of the Childhood Trauma Questionnaire short form; mCIS, modified Coronavirus Impact Scale; International Physical Activity Questionnaire (IPAQ) Code: 1 = low, 2 = moderate, 3 = high. Depression and anxiety measured using the Depression and Anxiety Subscales of the 21-Item Depression Anxiety Stress Scale.

The model was significant for depression [*F*(7, 398) = 46.29, *p* < 0.001] and accounted for 44.9% of the variance in depression scores. All three personality variables, childhood emotional abuse, and male sex emerged as significant predictors. The model for anxiety was also significant [*F*(7, 398) = 59.30, *p* < 0.001] and accounted for 51.1% of the variance in anxiety scores. Again, all three personality variables and childhood emotional abuse emerged as significant predictors. Neither COVID impacts nor physical activity levels accounted for unique variance in either model, when controlling for the other variables.

## 4 Discussion

This study adds to the literature (Debowska et al., 2020; Kaparounaki et al., 2020; Wang et al., 2020) showing that rates of clinically significant symptoms of anxiety and depression are alarmingly high in university students—a situation which was likely exacerbated by the COVID-19 pandemic (Sun et al., 2023). The current findings extend past work by systematically exploring risk and protective factors for mental health in this vulnerable group. Several noteworthy findings emerged from this research. First and foremost, we were able to tease apart variance in mental health outcomes attributable to alexithymia, SPS, AS, and childhood emotional abuse by considering these variables simultaneously, rather than looking at one variable at a time. This was important given the overlap between these variables, which may have confounded the results of earlier studies. We were able to show that each of the four variables accounted for unique variance in both depression and anxiety after controlling for several other potential risk/protective factors. These findings lend strong support to the conclusion that these variables represent key risk factors for these common mental disorders (Felitti et al., 1998; Taylor and Bagby, 2004; Liss et al., 2005; Deacon and Abramowitz, 2006; Carleton et al., 2009).

Separating the variance in mental health related to personality vs. childhood emotional abuse is important as these variables represent two distinct sets of factors thought to influence the development of between-person differences in the generation/experience (Smith et al., 2018) and regulation (Goldsmith et al., 2008; Canli et al., 2009) of emotions: genetic/epigenetic factors that influence the expression of (partially) heritable personality traits, and factors related to learning. With regard to the latter, Smith et al. (2018) emphasize the important role that early life experiences play in establishing cognitive habits (patterns of attention, motivation, and goal updating) that impact emotion processing and regulation. Some of these variables may *reinforce* innate personality traits, which would partially explain why past work has suggested that alexithymia, SPS, and AS may mediate links between childhood abuse and poor mental health (McLaughlin and Hatzenbuehler, 2009; Karaca Dinç et al., 2021; Yang et al., 2022). As an example, research suggests that early life adversity may change the threshold of limbic reactivity, and perceptual and cognitive appraisals of threat (Andersen et al., 1999; Thompson, 2011), potentially intensifying the expression of SPS and raising the risk of experiencing significant anxiety. The present finding that childhood abuse remains a significant predictor of both anxiety and depression even when key personality variables are held constant suggests that additional factors linked to childhood emotional abuse that are *unrelated* to the personality variables that we studied constitute unique risk factors for psychopathology. More research is needed to identify these factors and design interventions to address them effectively. In this regard, it is important to remember that children and adolescents frequently experience multiple forms of adversity, such as witnessing domestic violence or suffering other forms of maltreatment (Hamby et al., 2010). It is possible, then, that the variance accounted for by emotional abuse in the current study may be partly attributable to these factors.

The present findings extend other work suggesting that alexithymia, SPS, AS, and childhood emotional abuse are all associated with feeling more negatively impacted by the COVID-19 pandemic in one’s daily life (DeGrace et al., 2021; Gürsoy et al., 2021; Janiri et al., 2021; Güneş and Bulut, 2022; Iimura, 2022). We were able to show, for the first time, that SPS is the most important of the three personality variables in explaining individual differences in perceived COVID-19 impacts, but that these impacts were also felt more strongly by those reporting a history of childhood emotional abuse, those who engaged in higher levels of physical activity, and (to a lesser extent) females. More importantly, however, we were able to show that, once variance in personality and childhood emotional abuse was accounted for, information regarding pandemic-related impacts did not improve prediction of anxiety or depression in our university student sample. This complements the findings from Stanislawski et al. (2023) who found that, at the end of their year-long study, pandemic-related worries did not predict depression, anxiety, and/or PTSD in third-year medical students, whereas baseline psychological distress, resilience, and childhood emotional abuse did.

As a final, general comment, it is important to highlight the very high rates of alexithymia that were observed in our university student sample (see also, for example, Lyvers et al., 2014). This may reflect, in part, the idea that although alexithymia is generally considered to be a risk factor for poor mental health, it can also be exacerbated by it. Thus, there is evidence to suggest that both depression (Marchesi et al., 2008) and stress (Kristal and Krystal, 1988) can exacerbate symptoms of alexithymia.

### 4.1 Physical activity

The fact that current physical activity levels were not associated with mental health in university students was unexpected given previously documented benefits of physical activity on well-being (Wahid et al., 2016; Pearce et al., 2022). Equally surprising was the finding that current levels of physical activity were not more strongly correlated with the personality variables, or with exposure to childhood emotional abuse as (a) exercising less has been associated with scoring higher on alexithymia in males (Helmers and Mente, 1999), SPS in male and female university students (Yano et al., 2017), and AS in both females (Farris et al., 2019) and mixed samples (Moshier et al., 2015); and (b) exposure to emotional abuse in childhood has been linked to reduced participation in sport (Noel-London et al., 2021). Some discussion of these findings is warranted given these surprising results.

There are several possible reasons why the expected links between physical activity and these other variables were not apparent in the present sample. First, overarching stresses related to the context of the pandemic may have weakened these links. Second, almost all students surveyed were not only exercising less but also sitting more than they had been before the onset of the pandemic (see also Gallè et al., 2020; McCormack et al., 2021). Recent work suggests that time spent sitting is a strong predictor of mental health during the pandemic (Cheval et al., 2021; Runacres et al., 2021). Unfortunately, the IPAQ data regarding sitting time included many imprecise estimates (e.g., “most of the day”) and/or missing values; as such, we were not able to examine the relationship between sitting time and mental health.

A third additional factor relates to how accurately vigorous and moderate exercise and walking were assessed. Better activity data may have been obtained had we used the version of the short form in which individuals are asked to reflect on the last 7 days rather than on a “usual week,” although both versions are reported to be reliable (Craig et al., 2003). On the other hand, use of the long form of the IPAQ would have allowed us to assess links with specific *types* of physical activity (job-related; transportation; housework, house maintenance, caring for family; recreation, sport, and leisure-time; time spent sitting), rather than relying on a composite measure. It may also have been helpful to ask participants to indicate how much of their exercise took place outdoors (vs. indoors) given past work suggesting that (a) engaging in outdoor activity has a bigger impact on negative affect (anger, sadness, and stress) than engaging in indoor activity (Bowler et al., 2010; Dunton et al., 2015); and (b) levels of anxiety are lower in participants who spend more (vs. less) time exercising outdoors (Lesser and Nienhuis, 2020). Because data for the present study were collected in February and March when temperatures in Winnipeg tended to be well below freezing it seems likely that much of the physical activity our participants engaged in took place indoors.

A final reason physical activity was not strongly related to mental health, personality variables, or exposure to childhood emotional abuse in the present study could be that our sample included some individuals who were exercising at very high levels of intensity because they were dependent on (i.e., addicted to) exercise, and/or because they were elite or sub-elite athletes involved in competitive sport. It is possible that the relationship between physical activity and mental health, personality, and/or abuse is different in these subgroups than in less active individuals or in non-athletes.

### 4.2 Sex differences

Another set of results that warrant discussion relate to sex differences in mental health. Unlike other authors (see also Wang et al., 2020; Ballarotto et al., 2021; Sun et al., 2023), we saw only weak evidence that females were experiencing greater anxiety than males, and this sex difference disappeared when we controlled for other variables in our final regression. In addition, contrary to work carried out both before (Lim et al., 2018) and during the COVID-19 pandemic (Sun et al., 2023), females were not found to be more likely to report symptoms of depression than males. Indeed, in the regression predicting depression it was male sex that emerged as a risk factor. Together, these findings suggest that failing to account for the impact of personality factors such as SPS and AS—which we found to be more strongly expressed in females—may have introduced a confound in past research exploring sex differences in mental health. More research is needed to explore why male sex emerged as a vulnerability factor for depression in our study, but it is quite possible that males experience unique stressors and societal pressures that increase their risk of developing depression. For instance, traditional gender norms may discourage men from seeking help for mental health concerns, leading to underdiagnosis and undertreatment. Future researchers should also explore in more detail sex differences in the way that people alter their exercise routines in response to stressors, and what implications this might have for mental health.

In the sample as a whole, those who reported being more impacted by the pandemic (high mCIS score) also exercised more (high Total METs). This supports the view that exercise may be one way that people try to cope in times of stress. Indeed, high-intensity physical activity has been shown to produce long-lasting physiological changes that contribute to the benefits of exercise on cognitive and emotional functioning (Nakagawa et al., 2020). Recall, however, that females in the present study were more likely than males to have changed their exercise routine after the onset of the pandemic by increasing their moderate and vigorous activity levels. Females may have felt that making this change would help them cope. Due to the cross-sectional nature of the current study, it is impossible to say if these alterations had an immediate impact on females’ mental health. However, it is interesting to note that recent research exploring mental health trajectories during the pandemic suggests that, although females may have presented with more severe anxiety and depression early on, they also showed more rapid improvements in mental health over time (Fancourt et al., 2021). It is conceivable that the females in our study were more vulnerable to mental health problems prior to the pandemic (Kessler et al., 1994) but that they showed rapid, early improvements in their mental health as a result of changing their exercise routines when the pandemic began. This may partially explain why, when we tested them approximately 1 year into the pandemic, levels of anxiety and depression were fairly similar in males and females.

### 4.3 Limitations and future directions

There are a number of limitations to the present work. First, the study was carried out during the pandemic, when quarantine and social distancing rules were in place and institutional restrictions were placed on in-person testing. As such, we had to rely on the use of online data collection techniques, which may have negatively impacted how accurately certain constructs were measured. Although we used the Conscientious Responders Scale to try to screen out those exhibiting poor effort, the possibility remains that participants were less attentive and careful than they might have been had they completed the measures in-person. Having said that, recent evidence does suggest that online testing has not created a major threat to the generalizability of findings during the pandemic (Peyton et al., 2022).

Second, as noted earlier we have some concern about how accurately we were able to measure physical activity levels via the IPAQ. It is possible that participants in future studies would experience fewer problems with recall and/or respond more carefully if they were given the version of the IPAQ that refers to activities engaged in over the preceding 7 days, rather than the “usual week” version that was used here. In addition, future researchers may wish to supplement or replace self-report measures of activity with more objective indices, such as accelerometer recordings or daily activity diaries. This seems prudent given that correlations between self-report and these more objective measures of exercise have typically been found to be low (Craig et al., 2003).

Third, our measure of emotional abuse was very short, and we did not extract important information such as the onset, duration, or frequency of abuse. It is important to try to tease apart the impact that variables such as these have on development and mental health.

Finally, as noted earlier, the study design was cross-sectional, which limits one’s ability to make causal assumptions. More longitudinal research is needed to examine trajectories of anxiety and depressive symptoms during stressful times, not only in males and females but also in gender-diverse samples.

## 5 Conclusion

This study adds a number of important findings to the existing literature. First, it demonstrates that alexithymia, SPS, AS, and emotional abuse suffered in childhood each make a unique contribution to the prediction of mental health outcomes. Assessing variance uniquely attributable to each of these variables was important, given that past research has tended to ignore the fact that they are correlated with one another, with the result being that previously described links between these variables and other measures may have been confounded.

By comparing relationships between our study variables in males and females, we were able to comment on important sex differences in risk and protective factors for mental health in the context of a global pandemic. Given that females reported higher levels of SPS and AS in the present work, it will be especially important to control for these factors when examining mental health outcomes in males and females moving forward. It will also be important for clinicians to be mindful of this as they attempt to refine individualized treatment protocols for their clients.

As a final point, we want to remind the reader that the focus of the present investigation was on internalizing disorders. It will be important to extend this work in the future to determine risk and protective factors for disorders characterized by externalizing problems. Longitudinal studies may be particularly important in this regard, given recent evidence that chronically elevated levels of externalizing problems in childhood are more likely to have an “intrinsic emotional basis” (i.e., to be associated with temperamental emotionality, internalizing symptoms, and maternal mental distress) than externalizing problems that emerge in adolescence (Kjeldsen et al., 2021).

## Data availability statement

The datasets presented in this study can be found in online repositories. The names of the repository/repositories and accession number(s) can be found below: The dataset for this study can be found in the “Individual differences in mental health” repository at: https://doi.org/10.34990/FK2/EYYQOK.

## Ethics statement

The studies involving humans were approved by Research Ethics Board at the University of Manitoba (Fort Garry Campus). The studies were conducted in accordance with the local legislation and institutional requirements. The participants provided their written informed consent to participate in this study.

## Author contributions

CVL: Conceptualization, Data curation, Formal analysis, Investigation, Methodology, Project administration, Writing – original draft. LJ: Conceptualization, Formal analysis, Funding acquisition, Supervision, Writing – original draft.
